# The role of 5-HTergic neuron activation in the rapid antidepressant-like effects of hypidone hydrochloride (YL-0919) in mice

**DOI:** 10.3389/fphar.2024.1428485

**Published:** 2024-09-06

**Authors:** Guang-Xiang Li, Jiao-Zhao Yan, Sun-Rui Sun, Xiao-Juan Hou, Yong-Yu Yin, Yun-Feng Li

**Affiliations:** ^1^ Beijing Institute of Basic Medical Sciences, Beijing, China; ^2^ Beijing Institute of Pharmacology and Toxicology, Beijing Key Laboratory of Neuropsychopharmacology, Beijing, China; ^3^ Beijing Ditan Hospital Capital Medical University, Beijing, China; ^4^ Department of Postgraduate, Hebei North University, Zhangjiakou, China

**Keywords:** YL-0919, 5-HT, DRN, antidepressant, depression

## Abstract

**Introduction:**

Major depressive disorder (MDD) is a common and disabling mental health condition; the currently available treatments for MDD are insufficient to meet clinical needs due to their limited efficacy and slow onset of action. Hypidone hydrochloride (YL-0919) is a sigma-1 receptor agonist and a novel fast-acting antidepressant that is currently under clinical development.

**Methods:**

To further understand the fast-acting antidepressant activity of YL-0919, this study focused on the role of 5-HTergic neurons in the dorsal raphe nucleus (DRN) in mice. Using fiber photometry to assess neural activity *in vivo* and two behavioral assays (tail suspension test and forced swimming test) to evaluate antidepressant-like activity.

**Results:**

It was found that 3 or 7 days of YL-0919 treatment significantly activated serotonin (5-HT) neurons in the DRN and had significant antidepressant-like effects on mouse behaviors. Chemogenetic inhibition of 5-HTergic neurons in the DRN significantly blocked the antidepressant-like effect of YL-0919. In addition, YL-0919 treatment significantly increased the 5-HT levels in the prefrontal cortex (PFC). These changes were drastically different from those of the selective serotonin reuptake inhibitor (SSRI) fluoxetine, which suggested that the antidepressant-like effects of the two compounds were mechanistically different.

**Conclusion:**

Together, these results reveal a novel role of 5-HTergic neurons in the DRN in mediating the fast-acting antidepressant-like effects of YL-0919, revealing that these neurons are potential novel targets for the development of fast-acting antidepressants for the clinical management of MDD.

## Introduction

Major depressive disorder (MDD) is a severe mental illness; MDD is associated with a high risk of suicide (Alboni et al., 2017) and affects approximately 350 million people worldwide. Current available antidepressants have several limitations, including delayed onset of action (3–6 weeks), limited efficacy, and major side effects such as cognitive deficits (Li, 2020). Therefore, understanding the causes of depression and developing faster-acting and safer antidepressants are top priorities for the global medical research community.

Sigma-1 receptors are widely distributed in the central nervous system and play various crucial roles, including regulating intracellular calcium levels, influencing neurotransmission, and participating in cell apoptosis (Kourrich, 2017). Sigma-1 receptors are also believed to be associated with various neurological disorders and psychiatric conditions, making them a popular research topic (Mancuso and Navarro, 2017; Maurice and Goguadze, 2017; Jia et al., 2018). The literature suggests that sigma-1 receptor knockout can lead to depressive-like behaviors in mice, while different types of sigma-1 receptor agonists exert significant antidepressant-like effects (Moriguchi et al., 2015; Zhang et al., 2017; Li et al., 2023).

Hypidone hydrochloride (YL-0919) is a small chemical molecule and a selective sigma-1 receptor agonist, which was developed in our laboratory and is currently in phase II clinical trials (Ren et al., 2023). The initial research findings suggested that oral administration of YL-0919 exerts rapid antidepressant-like effects in both rodents (3–7 days) and rhesus macaques (9 days) (Ren et al., 2023). Additionally, YL-0919 appears to enhance neuroplasticity in the prefrontal cortex (PFC) and hippocampus by activating the BDNF-mTOR pathway (Ran et al., 2018; Sun et al., 2019; Yin et al., 2020; Zhang et al., 2022). However, the exact mechanisms underlying the antidepressant-like effects of YL-0919 are still not fully understood.

The development of depression is closely associated with the hypofunction of the brain’s monoamine neurotransmitters, such as dopamine (DA), norepinephrine (NE), and serotonin (5-HT) (Anderson et al., 1992). 5-HT has long been associated with emotional disorders (Meneses and Liy-Salmeron, 2012; Oslin et al., 2022) and is primarily produced by serotonergic neurons in the dorsal raphe nucleus (DRN) of the brain. The DRN has widespread connections to reward-related brain regions and is a significant source of serotonin in the forebrain (Malhi and Mann, 2018). 5-HT transmission is a key target for treating depression and other major psychiatric disorders (Vertes, 1991). Animal behavioral experiments and electrophysiological studies have shown that sigma-1 receptors increase 5-HT neurotransmission through various mechanisms, thereby exerting antidepressant effects. In a behavioral model, progesterone and BD-1047 (a sigma-1 receptor antagonist) counteracted the antidepressant-like effects induced by the combined administration of pramipexole and sertraline (Rogóz and Skuza, 2006). Additionally, electrophysiological experiments on 5-HTergic neurons provide more direct evidence that sigma-1 receptors enhance 5-HT neurotransmission in a rapid manner (Robichaud and Debonnel, 2004). Similar evidence was found in another study, where continuous treatment with SA-4503 (a sigma-1 receptor agonist) for 2 days increased the firing rate of 5-HTergic neurons in a dose-dependent manner (Lucas et al., 2008). These experiments suggest that sigma-1 receptor ligands have the potential to act as antidepressants with a rapid onset of action (Bermack and Debonnel, 2001). However, the role of the 5-HT system in the DRN in the antidepressant-like effects of YL-0919 remains unclear.

To directly test our hypothesis, we first used the sigma-1 receptor antagonist BD1047 to block the action of YL-0919 in the tail suspension test (TST) and DRN calcium signal recording experiments; the results further confirmed that YL-0919 functions as a sigma-1 receptor agonist *in vivo*. Subsequently, we employed the classical antidepressant fluoxetine for comparison and utilized fibre photometry recordings and chemogenetics techniques to investigate the role of the serotonergic system in the rapid-onset antidepressant effect of YL-0919.

## Materials and methods

### Animals

A total of 120 Male C57BL/6 mice (6–8 weeks old) were obtained from Beijing SPF Biotechnology (Beijing, China), allocated for *in vivo* fiber photometry calcium signaling experiments, immunofluorescence and ELISA. Forty Adult SERT-Cre mice (6–8 weeks old) were purchased from the Shanghai Model Organisms Center. These mice were used specifically to study 5-HT neurons. These animals were housed in groups of four to five mice per cage under controlled environmental conditions, with a 12-h light/dark cycle (lights on from 07:00 to 19:00) and *ad libitum* access to food and water. Initially, the mice were allowed to acclimate for 1 week to minimize stress and then were subjected to the baseline sucrose preference test. All the experimental animal procedures were approved by the Institutional Animal Care and Use Committee of the Beijing Institute of Basic Medical Sciences. Aseptic surgical procedures were performed under anesthesia, and all efforts were made to minimize animal suffering.

### Drugs and reagents

YL-0919 (white powder, purity ≥99.8%, #D5222-18-001) was obtained from Zhejiang Huahai Pharmaceutical Co., Ltd. BD1047 (Cat. No. HY-16996A) was obtained from Master of Bioactive Molecules. Fluoxetine was obtained from Sigma‒Aldrich (#56296-78-7). YL-0919 (2.5 mg/kg), BD1047 (4 mg/kg), and fluoxetine (10 mg/kg) were dissolved in saline as previously described (Manosso et al., 2013; Li, 2020; Liu et al., 2021). The dose of YL-0919 was chosen based on previous behavioral tests that assessed its antidepressant-like effects (Chen et al., 2018; Ran et al., 2018). Drugs were intragastrically administered at a dose of 10 mL/kg based on body weight.

### Stereotaxic surgery and virus infusion

Mice were anesthetized with pentobarbital (i.p., 80 mg/kg) and secured in a stereotaxic apparatus that was equipped with an electric heating pad. To expose the skull, an incision was made in the skin of the head of the mouse. Using the bregma point as a reference (zero point), the positions of the medial PFC (mPFC: AP +2.0, DV -2.55, ML +0.5) and the DRN (AP -4.3, DV -3.4, ML 0 with a 20° angle towards the midline in the coronal plane) were identified according to the literature.

Next, 33-gauge syringe needles (Hamilton) were used for virus delivery. The injection rates were set to 50 nL/min. After each injection, the needle was left in the brain for another 5 min before being slowly withdrawn to prevent virus leakage. The virus types, total injection volumes, and expression times are provided in Table 1.

**TABLE 1:** Information about the viruses mentioned in the article.

Designation	Source reference	Identifiers	Additional information
rAAV-hSyn-GCaMp6-WPREhGH pA	BrainVTA	PT-0145	Serotype: AAV2/9 Titre: 2.0 *10^12^ vg/ml Injection site: DRN Volume: 500 nL
rAAV-EF1α-DIO-GCaMp6-WPREhGH pA	BrainVTA	PT-0071	Serotype: AAV2/9 Titre: 2.0 *10^12^ vg/ml Injection site: DRN Volume: 500 nL
rAAV-EF1α-DIO-hM4D (Gi)-mCherry-WPREhGH pA	BrainVTA	PT-0043	Serotype: AAV2/9 Titre: 2.0 *10^12^ vg/ml Injection site: DRN Volume: 500 nL
PAAV-hSyn-5-HT3.0	BrainVTA	PT-4724	Serotype: AAV2/9 Titre: 2.0 *10^12^ vg/ml Injection site: mPFC Volume: 500 nL

### Fibre photometry

After the viral injection, the optical fibre cannula was inserted 0.1 mm above the viral injection site. Subsequently, the optical fibre was secured to the skull surface with adhesive, and finally, dental cement was applied for fixation. To increase stability, a skull screw with a diameter of 1 mm was implanted on the surface of the mouse skull. After the animals recovered from anesthesia, they were returned to their original cages to receive further care.

Fibre photometry signals were processed with custom-written MATLAB software. In summary, all the data were segmented based on behavioral events and the baseline phase. The formula for calculating the change in fluorescence (ΔF/F) was (F-F0)/F0, where F0 represents the baseline fluorescence signal averaged over a 10-second-long control time window. We initially segmented the data based on behavioral events. Subsequently, we calculated the average 5-HT and calcium signals in both the pre-and postphases (2/10 s). The time window was determined based on the duration of the behavioral bout. The response elicited during a behavior was calculated as the average ΔF/F during all the trials of a specific behavior.

### Chemogenomics

Chemogenetics involves the manipulation of designed and modified biomolecules through interactions with specifically designed small-molecule ligands (O'Connor et al., 2011; Cong et al., 2012; Fujimori and Conway, 2016). This method is used to either activate or inhibit cellular activities, facilitating the study of mechanisms that are related to intracellular signal transduction, gene transcription, and disease development. The DREADD agonist clozapine N-oxide (CNO; 5 mg/kg) was administered by intraperitoneal injection 1 hour before the behavioral experiments.

### Behavioural tests

#### Tail suspension test (TST)

In the TST, a segment of medical tape was first applied to the distal end of the mouse’s tail. Subsequently, the tape was secured with a dovetail clip, and the mouse was inverted and suspended in a tail suspension box (25 × 25 × 20 cm). For more detailed information on the procedure, please refer to a previous study (Liu et al., 2007). Each mouse was suspended and observed for 6 min, and the immobility time during the last 4 minutes was measured. As with the fibre photometry experiment, the mice were handled gently before the experiment. The fibre optic jumper was delicately inserted into the fibre on the mouse’s head before the suspension.

#### Forced swimming test (FST)

The FST was conducted following a previously described protocol (Armario, 2021). Briefly, each mouse was placed in a glass cylinder (height: 25 cm; diameter: 18.5 cm) that was filled with water (24°C ± 1°C) to a depth of 15 cm and allowed to remain there for 6 min. The water level was adjusted to force the animals to swim or float without touching the bottom. The immobility time during the last 4 min was measured, and the results that were obtained by two researchers who were blinded to the drug treatments were averaged. Immobility was defined as the time during which the mice floated in the water, with all limbs motionless or exhibiting only minimal movements that were necessary to keep their heads above the water. After the experiment, the mice were promptly dried with a towel and returned to their original cages.

#### Enzyme-linked immunosorbent assay (ELISA)

After being deeply anaesthetized with a large dose of sodium pentobarbital, the mice were swiftly decapitated, and their brains were immediately removed on ice. Using a mouse brain matrix (RWD), the DRNs were dissected and promptly transferred to liquid nitrogen, followed by storage at −80°C. For analysis, the samples were retrieved from the freezer and placed on ice, and detection was carried out according to the protocol of the ELISA kit (Shanghai Bio). Subsequently, the optical density values were read at 450 nm using a microplate reader.

#### Immunostaining

After anesthesia with a high dose of pentobarbital, the mice were subjected to intracardial perfusion with 0.9% physiological saline, followed by fixation with 4% paraformaldehyde (PFA) in PBS. After cryoprotection in 30% sucrose, the mouse brains were coronally sectioned (30 mm) using a cryostat (Leica CM 1900). For immunohistochemistry, the sections were blocked with PBS containing 0.3% Triton X-100% and 3% bovine serum albumin (BSA) and then incubated overnight at 4°C with a rabbit polyclonal antibody against TPH2/TH (1:400, Millipore; incubation for 20 h) and a chicken polyclonal antibody against c-FOS (1:400, Abcam). After washing, the sections were incubated at room temperature for 1 h with Cy3-conjugated goat anti-rabbit IgG (1:500; Jackson ImmunoResearch) and fluorescein-conjugated donkey anti-chicken IgG (1:500; Jackson ImmunoResearch). After washing with PBS, the sections were coverslipped with DAPI and 50% glycerol.

### Statistical analysis

All the data are presented as the mean ± standard error of the mean (SEM) and analyzed with GraphPad Prism 9.5 (GraphPad Software Inc., San Diego, CA, United States). The normality of all the data was assessed using a one-sample Kolmogorov‒Smirnov test, and homogeneity of variance was confirmed using Levene’s test. Unpaired or paired t tests were used for comparisons between the two groups. One-way analysis of variance (ANOVA) was used for comparisons among three or more groups of different animals. When applicable, two-way ANOVAs or two-way repeated-measures ANOVAs were employed to compare multiple groups under multiple testing conditions. Post hoc comparisons were conducted using the Tukey method. For all tests, *p* < 0.05 was considered statistically significant.

## Results

### The rapid antidepressant-like effects and DRN activation after YL-0919 treatment were blocked by the selective sigma-1 receptor antagonist BD1047

**FIGURE 1:**
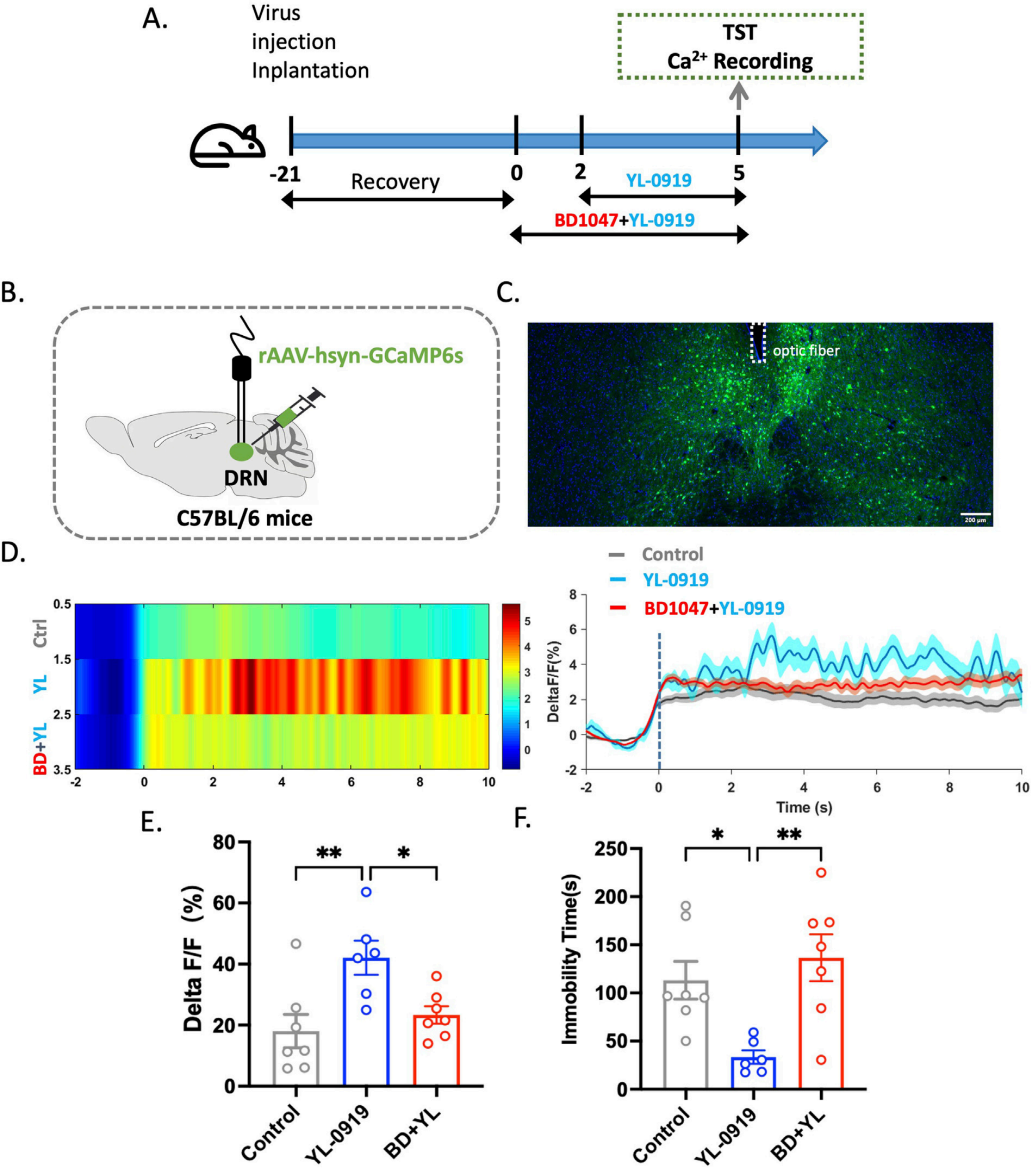
The rapid antidepressant-like effects of YL-0919 were significantly attenuated by BD1047. **(A)** Timeline of the experiments. **(B)** Schematic diagrams showing the virus injection and recording sites. **(C)** The specific expression of GCaMP6s (green) in DRN neurons. The dashed lines indicate optic fibre placement. **(D)** Graph showing changes in DRN calcium signaling. **(E)** Statistical analysis of the AUC. **(F)** The TST was conducted 24 h after administration on day 3. The data are presented as the means ± S.E.M., ^*^ *p* < 0.05, ^**^ *p* < 0.01. N = 6–7/group.

This study describes the methodology and results of a study that was conducted to evaluate the rapid antidepressant-like effects of YL-0919 using the TST and calcium signaling experiments. This study involved injecting the rAAV-hsyn-GCaMP6 virus into the DRN of mice, followed by surgical implantation of optical fibres (Figures 1B, C). After 21 days to allow expression of the genes carried by the virus, BD1047 and YL-0919 were administered on subsequent days. The TST and calcium signal experiments were then conducted 3 days after YL-0919 administration (Figure 1A). The results showed that YL-0919 significantly decreased the immobility duration of the mice (*F*_(2,17)_ = 7.4, *p =* 0.0273, Figure 1F) and enhanced calcium signaling within the DRN compared to the control (*F*_(2,17)_ = 6.7, *p =* 0.0067, Figure 1E). This effect was observed 3 days after YL-0919 administration (Figures 1D–F). In contrast, the BD1047-treated groups did not show significant differences in immobility duration (*F*_(2,17)_ = 7.4, *p =* 0.6618, Figure 1F) or calcium signaling compared to the control group (*F*_(2,17)_ = 6.7, *p =* 0.704, Figure 1E), indicating that the effects of YL-0919 were blocked by BD1047 (Figures 1D–F). These findings suggest that the activation of the sigma-1 receptor in the DRN mediates the rapid antidepressant-like effects of YL-0919. These findings support the hypothesis that YL-0919 has rapid antidepressant-like effects that are mediated through the sigma-1 receptor in the DRN.

### YL-0919 induced faster-onset effects on behavior and increased Ca^2+^ activity in the DRN

**FIGURE 2:**
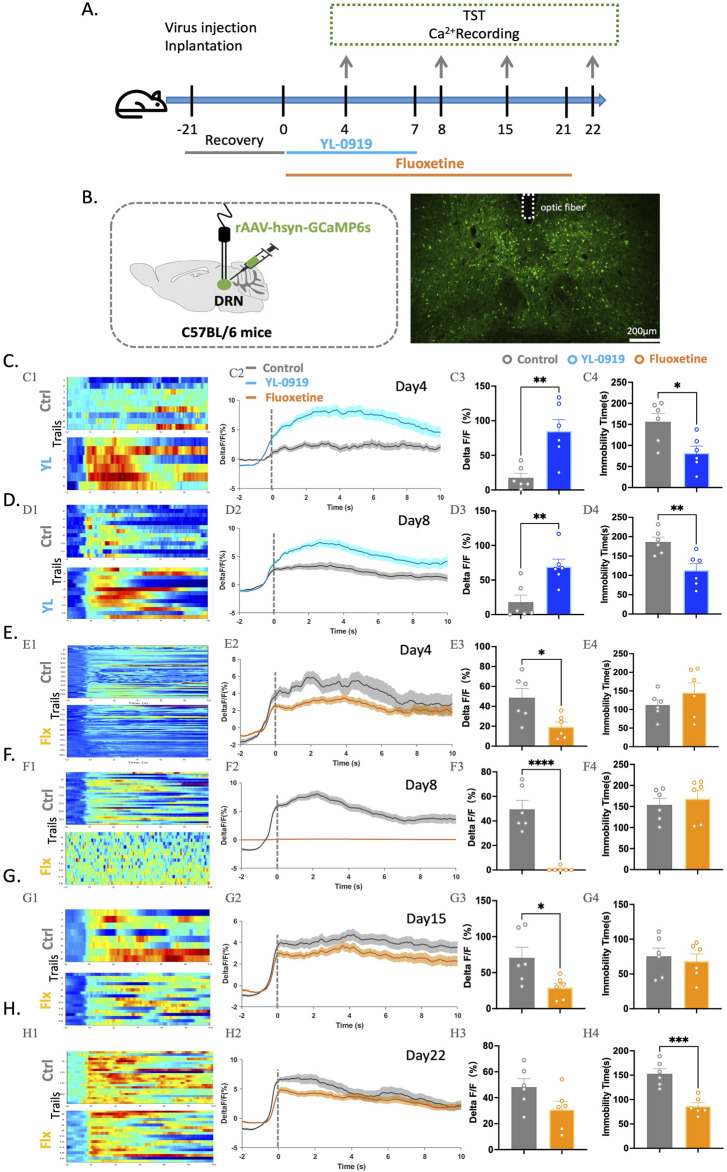
Contrasting effects of YL-0919 and fluoxetine on neuronal activation in the DRN of mice and immobility time in the TST. **(A)** Experimental timeline. **(B)** Schematic diagrams showing the virus injection and recording sites (left). The right panel displays the specific expression of GCaMP6s (green) in DRN neurons. The dashed lines indicate optic fibre placement. **(C–H)** (C1-H1) Heatmaps showing changes in Ca^2+^ signals in the DRN at different time points during the TST in the control group (top), YL-0919 group (bottom), and fluoxetine group (bottom). (C2-H2) Line graphs showing changes in Ca2+ signals in the DRN at different time points during the TST in the control group (grey line), YL-0919 group (blue line), and fluoxetine group (orange line). (C3-H3) Statistical results showing changes in Ca^2+^ signals in the DRN at different time points during the TST in the control group (grey), YL-0919 group (blue), and fluoxetine group (orange). (C4-H4) Statistical results showing immobility time at different time points during the TST in the control group (grey points), YL-0919 group (blue points), and fluoxetine group (orange points). The data are presented as the means ± S.E.M., ^*^ *p* < 0.05, ^**^ *p* < 0.01, ^***^ *p* < 0.001, ^****^ *p* < 0.0001. N = 6/group.

This study aimed to investigate the effects of YL-0919 on neuronal activity in the DRN using fibre optic recording experiments to observe Ca^2+^ activity during the TST. The experimental procedure involved injecting the rAAV-hsyn-GCaMP6 virus into the DRN and implanting ceramic ferrules for fibre optic recording (Figures 2B, C). After virus expression, the mice were treated with YL−0919 (2.5 mg/kg, i. g.) or fluoxetine (10 mg/kg, i. g.), which is a classical selective serotonin reuptake inhibitor (SSRI) antidepressant. Ca^2+^ signal recordings were performed on days 3, 7, 14, and 21 of drug treatment (Figure 2A).

The results showed that 3 days of YL-0919 treatment significantly reduced immobility time in the TST (*t*_(10)_ = 2.908, *p* = 0.0156) and increased Ca^2+^ signal activity in the DRN (*t*_(10)_ = 3.799, *p* = 0.0035, Figure 2C). Similar results were observed after 7 days (*t*_(10)_ = 3.447, *p* = 0.0063, Figure 2D) of YL-0919 treatment. In contrast, 3 days of fluoxetine treatment suppressed Ca^2+^ activity (*t*_(10)_ = 2.801, *p* = 0.0188, Figure 2E), and 7 days of fluoxetine treatment almost completely inhibited Ca^2+^ activity in the DRN (*t*_(10)_ = 6.709, *p* < 0.0001, Figure 2F). This inhibition began to be reversed after 14 days of fluoxetine treatment, and only after 21 days did fluoxetine reduce immobility time in the TST (*t*_(10)_ = 5.294, *p =* 0.0004) and ameliorate the inhibition of Ca^2+^ activity in the DRN (Figures 2G, H).

These findings suggest that both activation and reversal of inhibition of DRN neurons may exert antidepressant effects. The rapid activation of neurons in the DRN may play a crucial role in the rapid onset of the antidepressant-like effects of YL-0919.

### YL-0919 rapidly activated 5-HTergic neurons in the DRN

**FIGURE 3:**
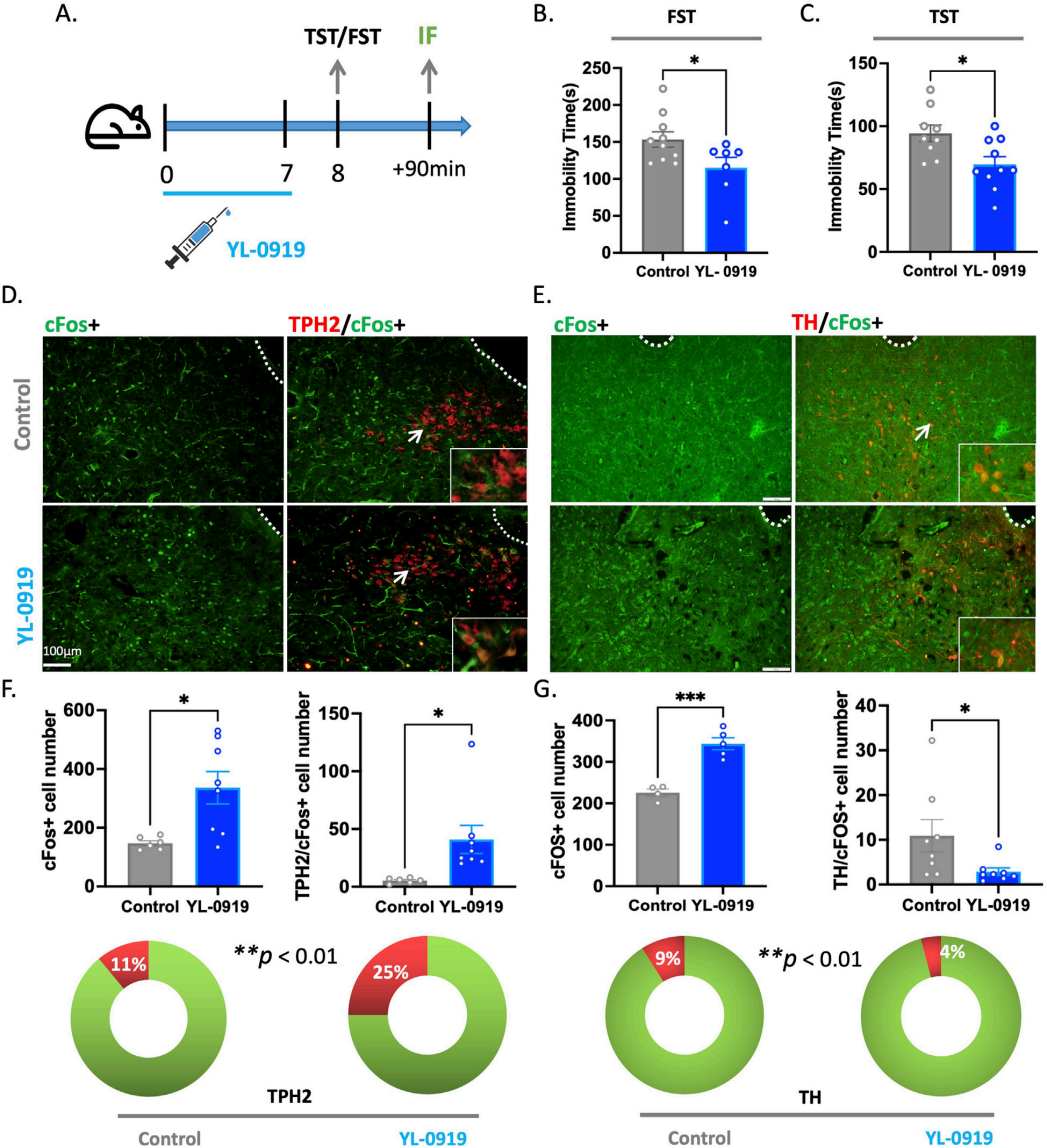
YL-0919 rapidly activated 5-HTergic neurons in the DRN. **(A)** Timeline of the experiments. **(B,C)** Immobility time of the YL-0919 group (blue) compared to that of the control group (grey) in the FST and TST (N = 7–10/group). **(D)** Expression of the c-Fos (green) protein in the immunofluorescence experiment, along with colocalization with TPH2 (red). **(E)** Expression of the c-Fos (green) protein in the immunofluorescence experiment, along with colocalization with TH (red). **(F,G)** Statistical results showing the number of c-Fos + cells (left), TPH2/c-Fos + cells (F-right), and TH/c-Fos + cells (G-right). The proportion of TPH2/c-Fos + cells (F-bottom). The data are presented as the means ± S.E.M.s, ^*^ *p* < 0.05, ^**^ *p* < 0.01, ^***^ *p* < 0.001. N = 4–8/group.

To further elucidate the specific type of neurons activated by YL-0919 in the DRN, we conducted the TST and FST in mice after 7 days of YL-0919 treatment (Figure 3A). Compared to the control group, YL-0919 significantly reduced immobility time in both the TST (*t*_(17)_ = 2.730, *p* = 0.0142) and FST (*t*_(15)_ = 2.242, *p* = 0.0405, Figures 3B, C) and increased c-Fos protein expression in the DRN (*t*_(12)_ = 2.927, *p* = 0.0127, Figures 3D, E). Furthermore, we performed immunofluorescence experiments to label activated neurons in the DRN with markers of different neuron types. The results showed a significant increase in the proportion of activated 5-HTergic neurons (*t*_(12)_ = 2.507, *p* = 0.0275, Figures 3D–F) and a decrease in the proportion of dopaminergic neurons among the activated neurons (*t*_(14)_ = 2.171, *p* = 0.0476, Figures 3E–G) in the YL-0919 group. This suggests that YL-0919 activates a higher proportion of 5-HTergic neurons and reduces the proportion of dopaminergic neurons among the activated neurons in the DRN. These findings reveal that the activation of 5-HTergic neurons in the DRN is a mechanism underlying the rapid antidepressant-like effects of YL-0919.

### Effects of YL-0919 on the Ca^2+^ activity of 5-HTergic neurons in Sert-Cre mice

**FIGURE 4:**
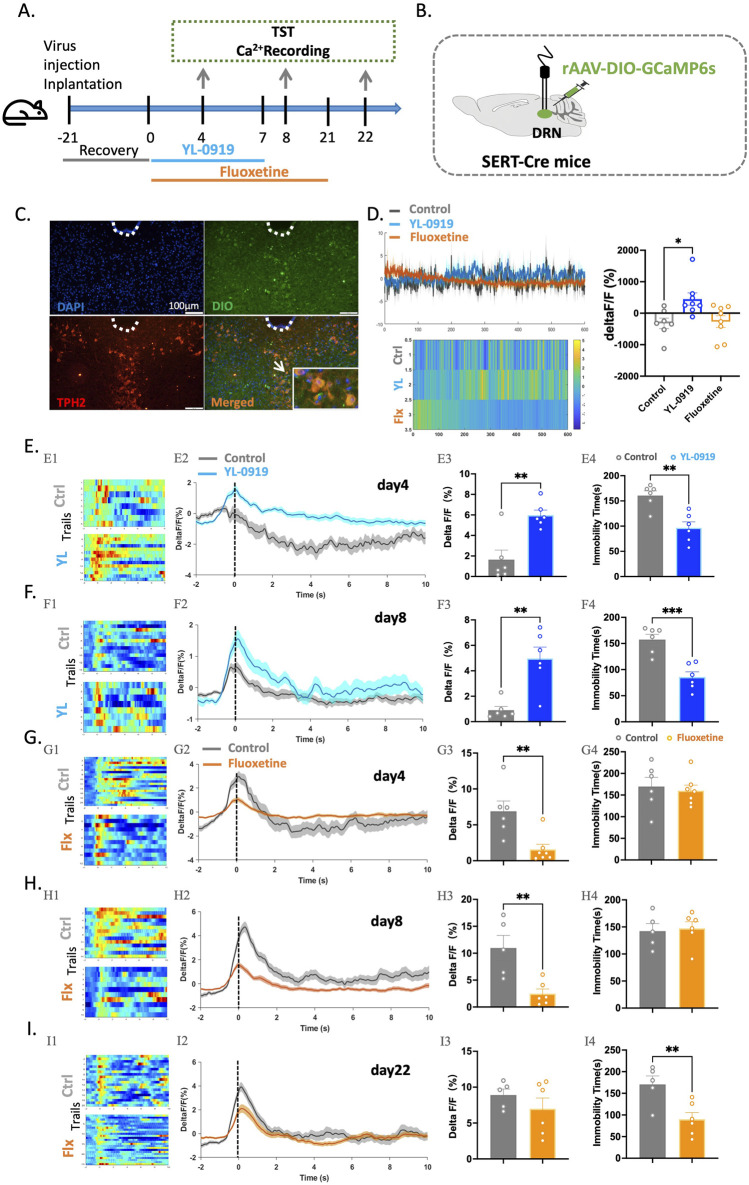
Compared to fluoxetine, YL-0919 exerted a rapid antidepressant effect and more effectively activated 5-HTergic neurons in the DRN of SERT-Cre mice. **(A)** Schematic diagrams showing the virus injection and recording sites. **(B)** Timeline of the experiments. **(C)** The specific expression of 5-HTergic neurons (green) in the DRN. **(D)** A single administration of YL-0919 activated 5-HTergic neurons, as shown in the heatmaps (left), line graphs (bottom), and statistical results (right) of the control group (grey), YL-0919 group (blue), and fluoxetine group (orange) N = 7–8/group. **(E,F)** Comparisons of changes in Ca^2+^ signals in the YL-0919 group (blue) and the control group (grey) on the 4th and 8th **(F)** days. **(G–I)** Comparisons of changes in Ca^2+^ signals between the fluoxetine group (orange) and the control group (grey) on the 4th **(G)**, 8th **(H)**, and 22nd **(I)** days, as well as changes in immobility time in the TST (E4-I4). See Figure 1. The data are presented as the means ± S.E.M.; ^*^ *p* < 0.05, ^**^ *p* < 0.01, ^***^ *p* < 0.001. N = 5–7/group.

Next, Sert-Cre mice were utilized to confirm whether 5-HTergic neurons in the DRN are indeed activated by YL-0919. First, the rAAV-DIO-GCaMP6s virus was injected into the DRN of mice, and fibre optic ferrules were implanted; experiments were conducted after 21 days of virus expression (Figures 4B, C). Similar to previous experiments, YL-0919 and fluoxetine were intraperitoneally administered for 3 days, 7 days, or 21 days, and then, mice were subjected to the TST and calcium signal recording of 5-HTergic neurons (Figure 4A).

In this study, calcium signals were recorded in the brains of freely moving mice over an extended period after a single dose of YL-0919 and fluoxetine. YL-0919 directly activated 5-HTergic neurons (*t*_(13)_ = 2.955, *p* = 0.0112), whereas fluoxetine did not have a similar effect (Figure 4D). Furthermore, after 3 days of YL-0919 treatment, a reduction in immobility time (*t*_(10)_ = 4.264, *p* = 0.0017) and a significant increase in calcium signals from 5-HTergic neurons (*t*_(10)_ = 4.113, *p* = 0.0021) were observed compared to those in the control group (Figure 4E). By the 8th day, YL-0919 continued to demonstrate an antidepressant-like effect (*t*_(10)_ = 4.989, *p* = 0.0005) and significantly activate 5-HTergic neurons (*t*_(10)_ = 4.299, *p* < 0.0016, Figure 4F). In contrast, the fluoxetine group showed significant inhibition of 5-HTergic neurons in the DRN during the TST after 3 (*t*_(11)_ = 3.479, *p* = 0.0052) and 7 days (*t*_(9)_ = 3.643, *p* = 0.0054) of treatment (Figures 4G, H). After 21 consecutive days, calcium signaling in the 5-HTergic neurons of mice recovered (*t*_(9)_ = 1.055, *p* = 0.3190, Figure 4I). Additionally, the immobility time in the fluoxetine group was significantly decreased (*t*_(9)_ = 3.264, *p* = 0.0098, Figure 4I).

### Chemogenetics-mediated inhibition of 5-HTergic neurons blocked the antidepressant-like effects of YL-0919

**FIGURE 5:**
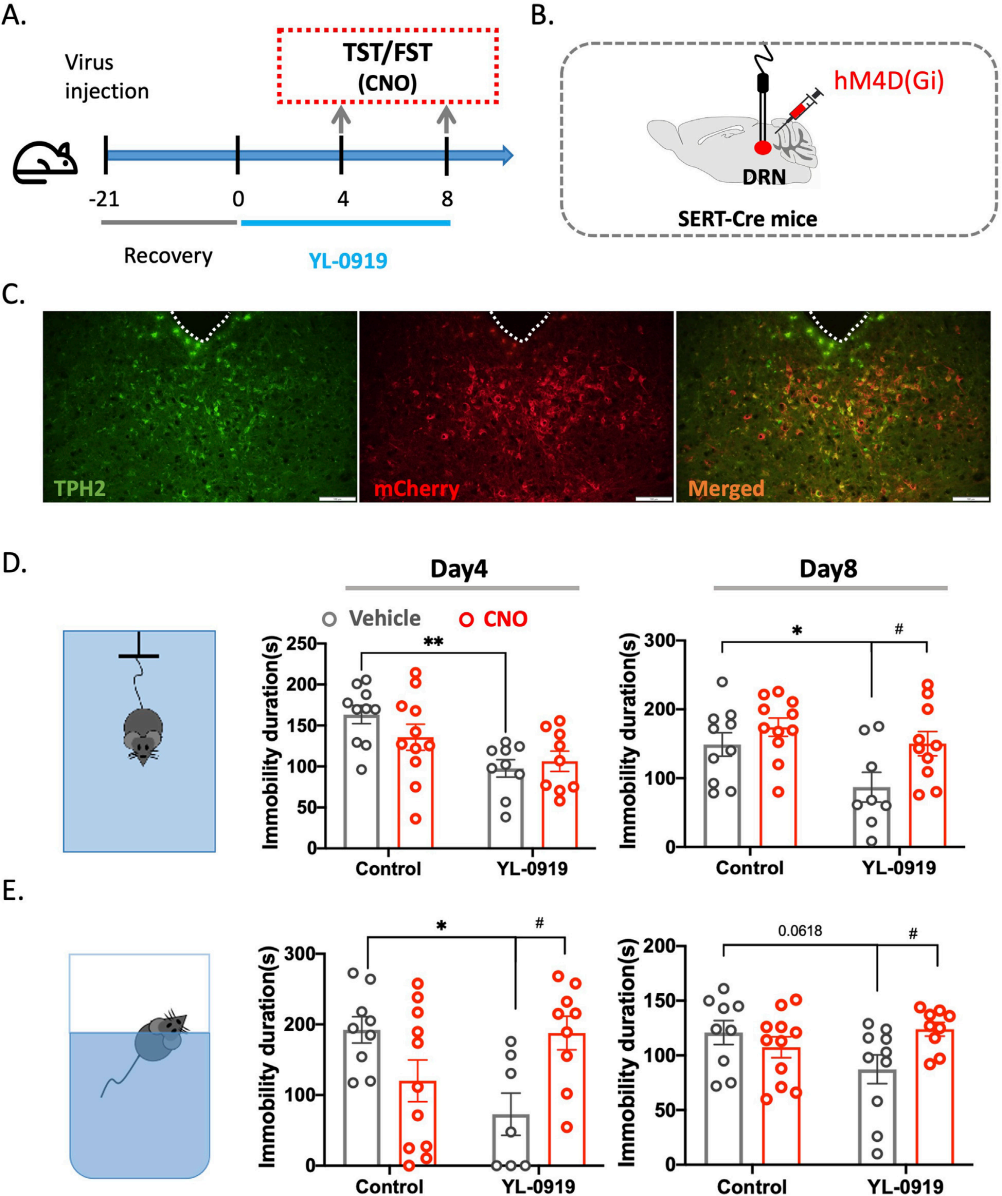
Inhibition of 5-HTergic neurons blocked the antidepressant-like activity of YL-0919 in the TST and FST using chemogenetics. **(A)** Timeline of the experiments. **(B)** Schematic diagrams showing virus injection. **(C)** Colabelling of viral expression (red) with TPH2 (green) to show colocalization (orange). **(D)** TST diagram (left), immobility time in the TST on the 4th (middle) and 8th (right) days of continuous YL-0919 administration. **(E)** FST diagram (left), immobility time in the FST on the 4th (middle) and 8th (right) days of continuous YL-0919 administration. The data are presented as the mean ± S.E.M., ^*^ *p* < 0.05, ^**^ *p* < 0.01, ^#^ *p* < 0.05, N = 8–11/group.

To confirm the critical role of 5-HTergic neurons in the antidepressant-like effects of YL-0919, we conducted a chemogenetic experiment using Sert-Cre mice. Essentially, a specific virus (rAAV-EF1α-DIO-hM4D (Gi)-mCherry) was injected into the DRN region of Sert-Cre mice. Once the virus was fully expressed, CNO was administered to temporarily inhibit the activity of 5-HTergic neurons (Figures 5A–C). Behavioral tests, including the TST and FST, were carried out 24 h after the final treatment of the 3-day or 7-day YL-0919 treatment regimen. CNO (5 mg/kg, i. p.) was administered 1 h before the behavioral tests.

ANOVA revealed that, compared to the control group, YL-0919 treatment for 3 days significantly reduced the immobility time of the mice in both the TST (*F*_(1, 35)_ = 13.13, *p =* 0.0025; Con + Vehicle vs. YL-0919+ Vehicle) and FST (*F*_(1, 32)_ = 12.26, *p =* 0.0258; Con + Vehicle vs. YL-0919+ Vehicle, Figures 5D, E). However, after the administration of CNO, there was a significant increase in immobility time in the FST (*F*_(1, 32)_ = 12.26, *p =* 0.0336; YL-0919 vs. YL-0919+CNO, Figures 5D, E). Furthermore, after 7 days of YL-0919 treatment, the immobility time in the TST significantly decreased (*F*_(1, 35)_ = 6.231, *p =* 0.0386; Con + Vehicle vs. YL-0919+ Vehicle), whereas in the CNO group, the immobility time in both the TST (*F*_(1, 35)_ = 6.231, *p =* 0.0349; YL-0919 + Vehicle vs. YL-0919+CNO) and the FST (*F*_(1, 35)_ = 5.697, *p =* 0.0392; YL-0919 + Vehicle vs. YL-0919+CNO) significantly increased (Figures 5D, E). This finding suggested that when 5-HTergic neurons were inhibited, the antidepressant-like effect of YL-0919 was lost, further confirming that the rapid activation of 5-HTergic neurons is the underlying mechanism responsible for the rapid onset of the antidepressant effects of YL-0919.

### YL-0919 increased the transmission of 5-HT in the mPFC

**FIGURE 6:**
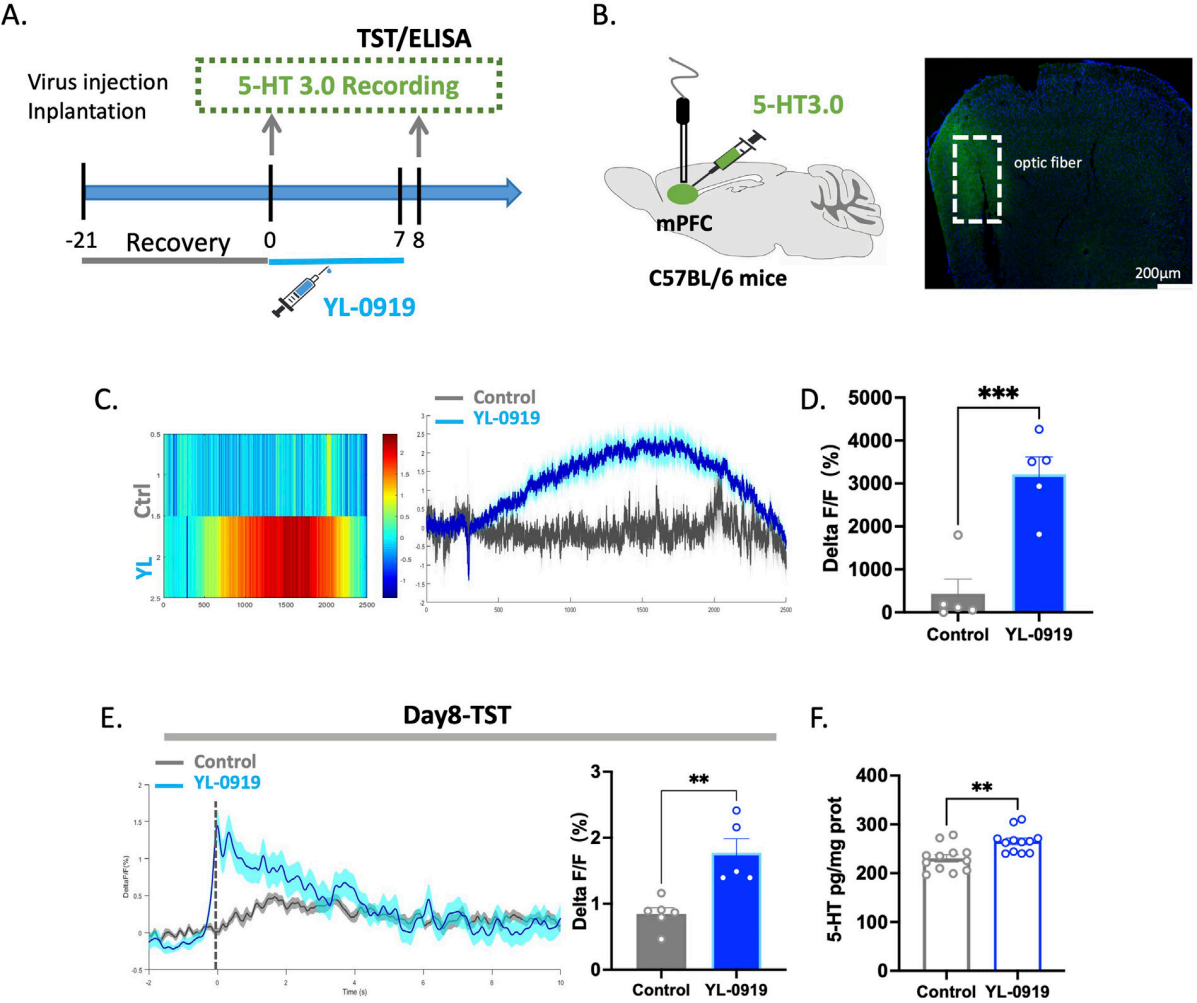
YL-0919 promoted the release of 5-HT within the mPFC. **(A)** Timeline of the experiments. **(B)** Schematic diagrams showing the virus injection and recording sites (left) The levels of 5-HT (green) in the mPFC (right). **(C)** Single-dose administration increased the 5-HT levels in the mPFC. From left to right, the figures represent the heatmap showing the change in the 5-HT levels, the curve graph. **(D)** Statistical results of changes in 5-HT Ca^2+^ signals between the control group and the YL-0919 group on the Single-dose treated. **(E)** The TST was conducted 24 h after administration on day 7. YL-0919 promoted 5-HT release in the mPFC. The figures showing the change in the 5-HT levels (left), and the statistical analysis of the 5-HT level (right). **(F)** ELISA experiments. The figure depicts the 5-HT levels in the mPFC. The data are presented as the means ± S.E.M., ^**^ *p* < 0.01, ^***^ *p* < 0.001, N = 5–6/group in the optical fiber recording experiment; N = 12/group in the ELISA experiment.

The mPFC and DRN have numerous projections between them and are closely involved in functions such as learning, emotions, mood, and cognitive function (Fukumoto et al., 2016). Previous research suggests that activity in the mPFC tends to decrease during depression (Seo et al., 2017). Previous experiments have confirmed the activation of 5-HTergic neurons by YL-0919. Therefore, 5-HT fluorescent probes were used to measure the 5-HT levels in the mPFC (Figures 6A, B). The results demonstrated that a single dose of YL-0919 significantly increased the levels of the neurotransmitter 5-HT in the mPFC compared to those in the control group during the spontaneous activity phase of the mice (*t*_(13)_ = 4.692, *p* = 0.0004, Figures 6C, D).

After 7 days of YL-0919 treatment, changes in the fluorescence of the 5-HT neurotransmitter in the mPFC of mice were monitored during the TST. Compared to those in the control group, there were higher levels of the neurotransmitter 5-HT in the mPFC of YL-0919-treated mice (*t*_(9)_ = 4.211, *p =* 0.0023, Figure 6E). To further assess the 5-HT levels in the mPFC, ELISA was performed after 7 days of treatment, and the results showed that YL-0919 increased the 5-HT levels in the mPFC of mice (*t*_(22)_ = 3.437, *p* = 0.0024, Figure 6F). This indicates that YL-0919 not only activates 5-HTergic neurons but also increases the release of the neurotransmitter 5-HT in relevant brain regions.

## Discussion

Consistent with previous findings that demonstrated the faster-onset antidepressant-like effects of YL-0919 in rodent and nonhuman primate models (Chen et al., 2013; Ran et al., 2018; Sun et al., 2019; Yin et al., 2020), this study further confirmed the rapid antidepressant-like effects of YL-0919 in mice after 24 h of treatment following either 3 or 7 days of dosing. Significantly, the rapid antidepressant-like effects of YL-0919 were negated upon prior administration of the selective sigma-1 receptor antagonist BD1047, indicating the direct involvement of the sigma-1 receptor in mediating the antidepressant-like effects of YL-0919. Furthermore, the activation of 5-HT neurons in the DRN and the augmentation of 5-HT transmission in the mPFC were associated with the behavioral effects of YL-0919, suggesting that 5-HT neuron activation in the DRN plays a pivotal role in mediating the faster-onset antidepressant-like effects of YL-0919.

In depression, the overactivity of somatodendritic 5-HT_1A_R autoreceptors in the DRN results in a reduced frequency of 5-HT neuron firing, decreased 5-HT levels in the synaptic cleft, and deactivation of postsynaptic 5-HT_1A_Rheter receptors (Stockmeier et al., 1998; Coplan et al., 2014; Seo et al., 2017). Fluoxetine, which is classified as a SSRI, exerts its therapeutic effects by promoting the gradual internalization and desensitization of presynaptic 5-HT_1A_ autoreceptors on the synaptic membrane. This may explain the delayed onset of action observed with fluoxetine in the clinic.

Heterodimer complexes play a crucial role in depression, especially those involving dimerization, such as 5-HT_1A_ receptors (Bermack et al., 2004; Ago et al., 2017). Recent research from our team revealed the presence of sigma-1 receptors that form complexes with 5-HT_1A_ receptors in the brain. Previous studies have reported that the sigma-1 receptor agonist SA-4503 can activate 5-HT neurons, and this effect can be blocked by sigma-1 receptor antagonists and 5-HT_1A_ receptor antagonists (Lucas et al., 2008). Our earlier results also indicated that YL-0919 can bind specifically to sigma-1 receptors, leading to a rapid antidepressant-like effect (Ren et al., 2023). GIRK channels, which are found within 5-HT neurons, are known as the primary effectors of 5-HT_1A_ autoreceptors. When 5-HT_1A_ autoreceptors are part of the sigma-1-5-HT_1A_ heterodimer complex, they may play a positive role in restoring the excitability of 5-HT neurons and facilitating the synthesis and release of 5-HT. Our early studies suggested that the formation of the sigma-1-5-HT_1A_ complex promotes the dissociation of 5-HT_1A_ receptors from Gγ/β subunits (unpublished data), leading to the rapid recovery of 5-HT neurons. Although the exact underlying mechanisms are not fully understood, these findings offer some insights into the rapid antidepressant-like effects of the sigma-1 receptor agonist YL-0919.

The fluoxetine-induced inhibition of the activity of 5-HTergic neurons in the DRN is gradually reversed with the duration of administration, which was consistent with earlier findings (Bétry et al., 2013). This disinhibition might be related to chronic fluoxetine-induced desensitization of 5-HT_1A_ autoreceptors located in the 5-HTergic neurons in the DRN, which was also the key to the emergence of antidepressant-like behavioral activity (Le Poul et al., 1995). Additionally, chronic administration of fluoxetine can significantly elevate the levels of 5-HT neurotransmitters in the mPFC, which might be another reason for its behavioral effects (Invernizzi et al., 1996). Moreover, recent studies have indicated that depression can increase the formation of the SERT-nNOS complex in the DRN. Disruption of this complex ameliorates the inhibition of 5-HT neurons in the DRN, leading to a decrease in the 5-HT concentration in the DRN and resulting in a rapid antidepressant-like effect (Sun et al., 2022). Therefore, further investigations into the mechanisms underlying the changes in 5-HTergic neurons in the DRN that lead to behavioral outcomes are needed in the future.

At the neural circuit level, the activation of 5-HT neurons in specific brain regions results in the release of 5-HT, which then leads to changes in the activity of downstream neurons. 5-HTergic neurons that are located in the DRN form complex synaptic connections with various nuclei throughout the brain via dense neural fibres. These connections receive input from regions such as the mPFC, hypothalamus, amygdala, lateral habenula, and ventral tegmental area, which originate from both the forebrain and limbic system. In addition, these 5-HTergic neurons project neural pathways back to these regions, forming complex neural circuits that perform diverse functions. Notably, the interaction between the mPFC and the DRN involves bidirectional projection (Zhou et al., 2017) The mPFC is known to be closely associated with rapid antidepressant responses (Xu et al., 2019; Price and Duman, 2020; Wang et al., 2022; Fan et al., 2023). Previous research observed a significant increase in mPFC activity during tests of depression-related behaviors, such as the TST and FST (Moda-Sava et al., 2019). Notably, ketamine enhances the activity of mPFC pyramidal neurons (Hare et al., 2020). In our study, we confirmed that YL-0919 can increase the 5-HT levels in the mPFC. Therefore, we believe that the DRN-mPFC circuit plays a crucial role in mediating the rapid antidepressant effects of YL-0919.

In the study, we used the tail suspension test (TST) and the forced swimming test (FST). These two tests simulate the state of despair in animals and are considered to reflect the depressive state to a certain extent. The forced swimming test (FST) is a commonly used behavioral test to assess the effects of antidepressant drugs. It simulates a state of despair in animals and evaluates depressive-like behavior by measuring the immobility time in water. In the test, animals are placed in a cylindrical container filled with water, where they cannot escape, forcing them to swim until they eventually become immobile. The effectiveness of antidepressants is usually indicated by a reduction in immobility time. This method enables researchers to effectively screen and evaluate the potential of novel antidepressant drugs, providing essential experimental evidence for drug development (Yang et al., 2018; Sun et al., 2022). Considering that there are significant differences in their sensitivity and performance between the TST and FST, we think that these two behavioral tests can complement and validate each other in our study. In addition, in other similar studies, both TST and FST are combined to ensure the smooth progress and reliability of the experiments (Sun et al., 2022). However, we are aware of the limitations in this study that warrant further investigations. Notably, in this study, we only used normal animals for the experiments and did not evaluate YL-0919 using a mouse depression model. Therefore, we are currently validating our experimental results across different mouse depression models to enhance the reliability of our findings. Besides, we utilized *in vivo* fiber photometry to record the activity of 5-HT neurons. To further refine the study of 5-HT, it is necessary to conduct *in vitro* experiments, such as patch-clamp recordings, to directly observe the effects of YL-0919 on 5-HT neurons. This would provide more direct evidence to support our experimental conclusions. Finally, further investigation into the rapid antidepressant mechanisms of the drug at the neural circuit level (DRN (5-HT)/mPFC(Glu)) is warranted.

In summary, the compelling evidence we have presented throughout this study strongly suggests that YL-0919, which is a nonmonoamine medication, exerts its rapid antidepressant effect by interacting with the monoamine system in the DRN. This complex interaction results in the modulation of both monoaminergic and nonmonoaminergic systems within specific brain regions, and this modulation is crucial for therapeutic effects. The intricate regulatory effects of YL-0919 on these brain regions and neurotransmitter systems are central to the rapid onset of its antidepressant activity. These results provide insight for the future development of antidepressant medications that extend beyond the monoamine hypothesis and focus on nonmonoamine receptors and circuit neuroscience for the discovery of fast-acting antidepressants.

## Data Availability

The raw data supporting the conclusions of this article will be made available by the authors, without undue reservation.
